# Dual-energy CT in oncology: technical principles and clinical prospects

**DOI:** 10.1186/1470-7330-15-S1-P51

**Published:** 2015-10-02

**Authors:** D Simons, M Kachelrieß, HP Schlemmer

**Affiliations:** 1 German Cancer Research Centre (DKFZ), Heidelberg, Germany

## Learning objectives

• Dual-energy CT (DECT) can amply contribute to a more efficient workflow in oncologic imaging.

• DECT has the potential to reduce the radiation dose and to replace classical dual-phase protocols.

• DECT improves oncologic imaging with improved tumour detection and opens up avenues for tissue differentiation and characterisation.

• DECT allows for optimised and repeatable therapy monitoring and for the integration of imaging and therapy in oncology.

• The potential of DECT in oncology has not been fully exploited yet.

## Content organisation

Key topics for advanced use of DECT in oncologic imaging are its use in primary diagnostics, therapy planning, tumour detection, tumour characterisation and grading, therapy monitoring and follow-up, reduction of radiation dose, and the integration of imaging and therapy. Of special interest in this case is DECT’s ability to obtain virtual unenhanced images, quantify contrast uptake, and obtain material-specific information. These capabilities open up avenues for tissue differentiation and, to some extent, tissue characterisation. The ongoing development of multi-energy (spectral) CT prototypes may further foster developments in the field.

## Conclusion

With its fast acquisition time, multiple application possibilities, wide availability, and continuous technical development, DECT can amply contribute to a more efficient workflow in oncologic imaging. Further refinements in data processing algorithms, modifications of scan protocols, and the ongoing development of photon-counting detector technologies may allow for even more detailed tissue characterisation. The potential benefit to oncologic settings will certainly be the subject of intense research and development in the future.

